# Differentiating Factitious from Malingered Symptomatology: the Development of a Psychometric Approach

**DOI:** 10.1007/s12207-017-9301-y

**Published:** 2017-11-09

**Authors:** Alfons van Impelen, Harald Merckelbach, Marko Jelicic, Isabella J. M. Niesten, Joost à Campo

**Affiliations:** 1 Forensic Psychology Section, Department of Clinical Psychological Science, Maastricht University, PO Box 616, 6200 MD Maastricht, The Netherlands; 2 Radix Forensic Psychiatric Hospital, Heerlen, The Netherlands

**Keywords:** Factitious disorder, Malingering, Feigning, Symptom validity, Response bias

## Abstract

Psychometric symptom validity assessment is becoming increasingly part and parcel of psychological and neuropsychological assessments. An unresolved and rarely addressed issue concerns the differentiation between factitious and malingered symptom presentations: present-day symptom validity tests can assess *whether* an examinee presents with noncredible symptomatology, but not *why* an examinee does so. We explored this issue by developing the *Symptom and Disposition Interview* (SDI); a symptom validity test that incorporates strategies intended to gauge internal incentives associated with factitious disorder. The merits of the SDI were explored and compared to a traditional symptom validity test (the *Structured Inventory of Malingered Symptomatology*) in two analogue studies, each with factitious and malingering conditions (*n* = 24–30 per condition) and a clinical control group (*n* = 34, *n* = 40). Overall, the results were positive: The SDI was as effective in detecting feigned symptom presentations as a traditional symptom validity test and superior in differentiating factitious from malingered symptom presentations. We conclude that the SDI is not ready for clinical use, but that psychometric approaches to the assessment of factitious symptomatology, such as the SDI, appear sufficiently promising to warrant future research.

## Differentiating Factitious Symptomatology from Malingered Symptomatology

Psychiatric, psychological, and neuropsychological evaluations are arguably incomplete if they do not entertain the possibility that examinees may present with feigned symptomatology. Indeed, the vast majority of North American neuropsychologists hold assessment of feigned symptomatology to be “mandatory in forensic evaluations and at least desirable in clinical evaluations” (Martin, Schroeder, & Odland, 2015, p. 741). Assessment of feigned symptomatology is generally achieved through dedicated validity tests, which fall into two categories that each target a relatively independent dimension of assessment; *performance validity tests* (PVTs) assess the credibility of performance on cognitive tests, and *symptom validity tests* (SVTs) assess the credibility of symptom report (Larrabee, 2012). The effectiveness of validity tests to detect feigned symptomatology is well-established to the point that their use is recommended by organizations such as the Association for Scientific Advancement in Psychological Injury and Law (Bush, Heilbronner, & Ruff, 2014), the British Psychological Society (2009), and the Institute of Medicine (2015). Once feigned symptomatology has been established, “the next and equally important goal involves a careful assessment of multifaceted motivations for feigning” (Merten & Rogers, 2017, p. 101). However, while current validity tests function well in detecting feigned symptom presentations, they do not address potential motivations for such symptom presentations.

Historically, validity tests were considered to be tools to detect malingering; a notion that was abandoned around the turn of the millennium in favor of the idea that validity tests capture noncredible symptom presentations, but not the underlying reasons for such presentations (Merten & Merckelbach, 2013; van Impelen, Merckelbach, Jelicic, & Merten, 2014). Still, because validity tests originated from attempts to standardize the assessment of malingering (see Nies & Sweet, 1994) and malingering is driven by external incentives—which, by virtue of being external, can often be identified independently of patients’ self-report—there are no validity tests that address the motivations (external or internal) behind feigned symptom presentations.

Malingering is defined by the *Diagnostic and Statistical Manual of Mental Disorders* (DSM-5; American Psychiatric Association, 2013) as “the intentional production of false or grossly exaggerated physical or psychological symptoms, motivated by external incentives” (p. 726). To substantiate that a symptom presentation is malingered, three features of malingering must be established: these are (a) that the presented symptomatology is feigned; (b) that the feigning is intentional; and (c) that the intention is motivated by external incentives, such as avoiding work or duty, evading responsibility, or obtaining financial gain.

Another condition that may underlie feigned symptomatology is factitious disorder, which—in contrast to malingering—is considered to be a genuine mental disorder (American Psychiatric Association, 2013). The determination of factitious disorder is similar to the detection of malingering, as both require the identification of (a) symptomatology that is feigned and (b) feigning that is intentional. The difference between factitious disorder and malingering lies in the third criterion, (c) the motivation behind intentional feigning: Whereas malingered symptom presentations are motivated by external incentives, factitious symptom presentations are motivated by internal incentives, such as seeking nurturance and sympathy for being ill; this has also been referred to as the need to “assume the sick role” (American Psychiatric Association, 2013, p. 727). To our knowledge, all validity tests bear on (a) symptomatology that is feigned; some validity tests—e.g., certain PVTs; see below—address (b) intentional feigning; yet no validity test taps (c) motivation by internal incentives.

Currently, the only validity tests that might contribute data to the assessment of intentionality of feigning are PVTs that can identify below-chance performance. The forced-choice format of such PVTs allows for the calculation of the probability that a given score is obtained by chance: Test performances far below chance level imply deliberate avoidance of correct answers and are therefore equated with intentional under-performance (e.g., see Binder, Larrabee, & Millis, 2014). However, below-chance performance is a fairly unsophisticated form of feigning that is relatively rare compared with more subtle feigned symptom presentations (Greve, Binder, & Bianchini, 2009; van Impelen, Jelicic, Otgaar, & Merckelbach, 2017). Moreover, being cognitive tasks, PVTs are ill-suited to address psychological constructs such as “incentives” and “motivation”. SVTs, on the other hand, take the form of inventories, such as interviews and questionnaires of psychological symptoms and cognitive complaints. Examples include the Multiphasic Personality Inventory-2 Restructured Form (MMPI-2-RF; Ben-Porath & Tellegen, 2008) and the Personality Assessment Inventory (PAI; Morey, 1991). The format of SVTs is suitable to be modified to include assessment of incentives related to feigned symptom presentations. For example, Van Egmond and Kummeling (2002; see also Van Egmond, Kummeling, & van Balkom, 2005) developed a self-report checklist to explore incentives that patients anticipate when they enter treatment.

Although the presence of internal incentives to feign symptoms was deleted from the diagnostic criteria of factitious disorder when the DSM transitioned to its 5th edition, it has been retained as the main distinguishing feature between factitious disorder and malingering (American Psychiatric Association, 2013, p. 727). The removal of the internal incentives criterion likely reflects both the difficulties in determining the presence or absence of such incentives and the scarcity of research into the standardized assessment of factitious presentations. Virtually all published research into the assessment of factitious presentations is centered on individual cases (Bass & Halligan, 2014).

To our knowledge, the only systematic data on differentiating factitious from malingered presentations was gathered by Rogers and colleagues, who compared the SVT profiles of patients with factitious disorder with those of suspected malingerers; they found no consistent differences between validity profiles (i.e., Structured Interview of Reported Symptoms profiles; Rogers, Bagby, & Dickens, 1992; see also Rogers, Bagby, & Vincent, 1994). Rogers, Jackson, and Kaminski (2005) conducted the only experimental study to date that investigated the capacity of validity tests to differentiate between factitious and malingered presentations. More specifically, Rogers et al. (2005) administered a multiscale inventory of psychopathology with embedded validity scales (the PAI) and a symptom validity test (the Structured Inventory of Malingered Symptomatology, SIMS; Smith & Burger, 1997) to four groups of doctoral students; two groups were asked to simulate factitious symptom presentations (i.e., with *dependent* and *demanding* interpersonal styles, respectively), one group was asked to simulate malingered disability, and one group to respond honestly. Discriminant functions aside, only the PAI Defensiveness Index and a newly developed SIMS index (AF minus N) evidenced capacity to distinguish between factitious and malingered presentations (Rogers et al., 2005). Specifically, Rogers and colleagues (2005) found that those instructed to present with factitious symptomatology scored significantly higher on an index that subtracts the *Neurological impairment* (N) from the *Affective disorders* (AF) SIMS scale score.

The lack of psychometric measures of internal incentives can be seen as a deficiency of contemporary symptom validity assessment, but the lack of contemporary symptom validity assessment in the diagnosis of factitious disorder is perhaps more alarming: Psychometric symptom validity assessment was not mentioned among the factors leading to the diagnosis of factitious disorder in any of the 455 cases that were included in a recent systematic review of the scholarly literature on factitious disorder (Yates & Feldman, 2016). It may be that SVTs remained unmentioned in the review by Yates and Feldman (2016) because only cases with primarily physical symptoms were included. SVTs are typically used in psychological and neuropsychological evaluations and less so in medical domains that deal mainly with objective physical symptoms. However, given that SVTs are noninvasive and efficient, their informational value extends to practically any situation in which patients present with symptoms that are not readily or reliably assessed via physical examinations, including many cases reviewed by Yates and Feldman (2016).

Important goals of the current studies are to explore the potential of psychometric assessment of factitious symptom presentations and to address the lack of systematic research into the differentiation of factitious and malingered presentations. We endeavored to achieve these objectives through developing and testing the *Symptom and Disposition Interview* (SDI). We specifically designed the SDI to screen for feigned symptomatology *and* to gauge potential internal incentives for feigning (i.e., the need to assume the sick role). The utility of the SDI was examined in two studies using an analogue design with three experimental conditions, augmented with a clinical control group. The SIMS was employed as comparison for the ability of the SDI to detect feigned symptomatology (convergent validity) and to distinguish factitious from malingered symptom presentations (discriminant validity). Rather than serving to finalize the SDI for clinical use, the present studies function as exploratory steppingstones to improve the systematic assessment of factitious and malingered presentations.

## Study 1

### Method

Study 1 used an analogue design with three experimental conditions, a nonclinical control condition, and a clinical control group. The three experimental conditions addressed presentations of factitious disorder, illness anxiety, and malingering, respectively. The nonclinical control condition was included to assess the susceptibility of the SDI to genuine symptomatology: This was realized by comparing the participants in the nonclinical control condition with the clinical control group. To avoid potential cases of feigning in the clinical control group, participants scoring > 16 on the SIMS were treated as a separate group for all statistical analyses.

At the outset of study 1, we had four hypotheses. First, we expected the accuracy of the SDI in classifying feigned symptoms presentations (i.e., the experimental conditions) to be satisfactory, comparable to that of the SIMS. Second, because the SDI includes a scale targeting the need to assume the sick role, we predicted that the SDI, but not the SIMS, would be able to differentiate between factitious symptom presentations and malingered symptom presentations. Third, we surmised that the SDI, which has a scale tapping somatic sensitivity and illness anxiety, would distinguish feigned illness anxiety from malingered symptom presentations. And fourth, because the SDI, in contrast to the SIMS, includes a scale containing items alluding to common, credible symptomatology, we expected the SDI Unlikely Symptom scale (which is a symptom validity scale; see below) to be less sensitive to authentic symptomatology than the SIMS. In other words, we anticipated that differences in SDI scores of clinical and nonclinical controls would mostly manifest in regular clinical items, not in unlikely or noncredible clinical items.

### Participants

Participants for the three experimental conditions and the nonclinical control condition (*n* = 24 per condition) were recruited at the faculty of Psychology and Neuroscience of Maastricht University (both graduate and undergraduate students were allowed to participate). The great majority of this portion of the sample was female (90%; *n* = 86) and Caucasian (94%; *n* = 90). The mean age was 23.3 years (*SD* = 7.5; range 17–70). Because the study relied heavily on written materials, only native speakers of Dutch were eligible for participation. A history of severe mental illness was employed as exclusion criterion, but no participants disclosed such a history.

Participants for the clinical control group (*n* = 34) were recruited at three outpatient units of Radix Forensic Psychiatric Hospital, located in the Netherlands. This group was predominantly Caucasian (85%; *n* = 29), consisted almost exclusively of males (91%; *n* = 31), and had a mean age of 38.9 years (*SD* = 10.4; range 22–57 years). Patients at Radix are in treatment for delinquent behavior, ranging from repeated misdemeanors to serious felonies, but not homicide. Typical delinquencies included drug offenses, crimes against property (e.g., burglary and robbery), and offenses against persons (e.g., assault, battery, and sexual assault). The most frequent types of psychopathology among patients were substance disorders (63%) and personality disorders (53%). Other forms of psychopathology included affective disorders (e.g., bipolar and depressive), thought disorders (e.g., schizophrenia spectrum), neurodevelopmental disorders (e.g., autism spectrum, intellectual disability), and posttraumatic stress disorder. Comorbid symptomatology was common and generally involved personality and substance disorders.

### Measures

In addition to the SDI and the SIMS, which are described in more detail below, participants completed one other symptom validity test; the *Miller Forensic Assessment of Symptoms Test* (M-FAST; Miller, 2001; Cronbach’s alpha = .93). The M-FAST is a brief structured interview that inquires about various rare, unusual, and extreme psychiatric symptoms. With permission of its publisher, Psychological Assessment Resources, we translated the M-FAST into Dutch and included it in the current studies to investigate its psychometric properties. The M-FAST data were not used in the analyses presented in this report.

#### Symptom and Disposition Interview

The *Symptom and Disposition Interview* (SDI) is a newly developed screen for feigned symptomatology and internal incentives for feigning. The development of the SDI commenced with the first author conceiving four scales that, at least theoretically, appear useful for symptom validity assessment; a symptom validity scale, a regular clinical scale, a scale addressing the need to assume the sick role, and a scale covering illness anxiety and sensitivity to somatoform symptomatology. For each scale, a superfluous number of potential items were drafted. Through multiple discussions with the other authors, items were selected and revised, and several new items were created. The version of the SDI that was used in the present studies consists of 42 items constituting four scales: Unlikely Symptoms (US, 14 items), Common Symptoms (CS, 7 items), Sick Role (SR, 14 items), and Illness Anxiety (IA, 7 items). The scale scores are calculated by summation, as is the total score, which is taken to be an overall indicator of feigned psychopathology. The language of the SDI is Dutch; the sample items given throughout the text below are translations of the original Dutch versions.

The Unlikely Symptoms (US) scale comprises rationally derived items based on well-established detection strategies for feigned symptomatology, such as *improbable symptoms* and *symptom combinations* (Rogers, 2008b). Items consist of a question followed by three answer-alternatives: *often* (2 points), *sometimes* (1 point), and *never* (0 points). Examples are “When you are suffering from anxiety or panic, how often do you get overly sensitive to light?” and “When you cannot remember something, how often do you develop a headache?”.

The Common Symptoms (CS) scale contains items that inquire about different general symptoms whose mild variants occur frequently in nonclinical populations and whose severe variants are common in clinical populations. The answer and scoring format of CS items is equivalent to that of the US items. The CS items serve a fourfold purpose: (1) they provide room to report genuine symptomatology; (2) they aid in the detection of feigned symptomatology because they tap different types of pathology (as per the *indiscriminant symptom endorsement* detection strategy) as well as the frequency of symptoms (per the *symptom severity* strategy; Rogers, 2008b); (3) they provide an indication of symptom underreporting if patients largely deny any symptom manifestation; and (4) they veil the purpose of the SDI as an instrument to detect feigned symptomatology. Sample items are “How often do you suffer from depressive thoughts or feelings?” and “How often do you suffer from compulsive thoughts?”.

The Sick Role (SR) scale includes items that query the willingness to engage in patient-related activities, such as participating in patient support groups or scientific studies and undergoing treatment or diagnostic procedures, even if these are unpleasant or have serious side effects. Because Rogers et al.’s (2005) analogue study found simulators of factitious presentations to endorse items that allude to low self-worth and resentment toward others, we decided to also include such items in the SR scale (for example, “How often do you have the feeling that you are treated less well than other people?”). The answer and scoring format of the majority of SR items is divergent; 10 SR items provide only two answer alternatives, *yes* and *no*, which are scored as 2 or 0 points depending on the question. Typical SR items are “Are you prepared to take medication for your symptoms even if it would have serious side effects? Yes or no?”; “Are you willing to participate in scientific studies into new treatments for your symptoms? Yes or no?”; and “How often are you in need of psychological support? Often, sometimes, or never?”.

The Illness Anxiety (IA) scale consists of items covering distress over potential pathology and (self-perceived) sensitivity to somatic symptoms. The answer and scoring format mirrors that of the US and CS items. We included illness anxiety and somatic sensitivity items because they may aid in the detection of internal incentives or motivations for feigned symptomatology. The internal motivations that drive feigned symptom presentations in factitious disorder are similar to the internal motivations that underlie unconsciously distorted symptom presentations in *somatic symptom and related disorders* (including *illness anxiety*; see DSM-5; American Psychiatric Association, 2013, p. 309). Some authors (e.g., Krahn, Bostwick, & Stonnington, 2008) have even proposed to reclassify factitious disorder as a subtype of somatoform disorders, the argument being that factitious patients exaggerate their symptoms so as to convey to clinicians their belief that they are ill. Therefore, we reasoned that an assessment that is sensitive to somatoform symptomatology will also be sensitive to internal incentives or motivations for feigned symptomatology. Examples of IA items are “How often do you worry about your physical or mental well-being?” and “How often do you suffer from mildly allergic reactions?”.

#### Structured Inventory of Malingered Symptomatology

The *Structured Inventory of Malingered Symptomatology* (SIMS; Smith & Burger, 1997; see Merckelbach & Smith, 2003 for the Dutch version, Cronbach’s *α* = .72) is a screen for feigned symptomatology. It consists of 75 true-false statements that refer to improbable and atypical symptoms of affective disorders, psychosis, neurological impairment, memory dysfunction, and low intelligence. The total number of endorsed statements serves as an indicator of feigned symptomatology; scores above 16 warrant further assessment of symptom validity (van Impelenet al., 2014).

We employed the SIMS because it is a well-researched instrument with respectable psychometric properties, which therefore could fulfill multiple functions: (a) to compare the accuracy of the SDI in detecting feigned symptomatology with; (b) to guarantee the validity of the clinical control group by identifying possible cases of feigning; and (c) to gauge the effectiveness of our experimental manipulation in inducing feigned symptomatology through comparison with previous research (e.g., Rogers et al., 2005; van Impelen et al., 2014). Rogers et al. (2005) found that subtracting the *Neurological impairment* (N) scale from the *Affective disorders* (AF) scale of the SIMS resulted in an index that discriminated well between an experimental malingering condition, on the one hand, and two experimental factitious conditions, on the other hand. We included the SIMS AF-N index in our analyses to see if we could replicate the findings of Rogers et al. (2005).

### Procedure

Approval to commence the study was obtained from Radix Forensic Psychiatric Hospital as well as the ethics committee of the Faculty of Psychology and Neuroscience of Maastricht University. Data were gathered exclusively for research purposes and were not available to anyone but the researchers. With the exception of the informed consent, none of the study materials were signed with information other than participant numbers, thus guaranteeing the anonymity of the data that participants produced.

Nonclinical participants were quasi-randomly assigned to one of four conditions, which are outlined below; factitious, illness anxiety, malingering, and control. Both clinical and nonclinical participants were tested individually and in the presence of one of three researchers. The three researchers were advanced undergraduate psychology students who had completed supervised training in test administration and clinical interviewing. All information and instructions were conveyed via sets of written material, which contained the information facilitating informed consent, the instructions, and, in the experimental and nonclinical control conditions, a description of the experimental scenario. To increase the external validity of the experimental conditions, participants (including nonclinical controls) received instructional sets 2 days prior to the test session, leaving them with ample time to prepare themselves for participation in any way they saw fit. Exclusive employment of written materials allowed the experimenters to be blind to the conditions that participants were assigned to and hence avoid potential experimenter effects. Possible order effects were offset by administering all measures (i.e., SDI, SIMS, M-FAST) in counterbalanced order within each group. The total session time was around 50 min.

To ensure that the experimental materials were intelligible, plausible, and easy to identify with, we used the actually existing psychological support unit of Maastricht University as the setting for the scenarios and instructions (see below). After completion of the measures, participants were presented with several manipulation checks in the form of questions examining recall and compliance with the experimental manipulations. To safeguard the experimental procedures, participants were not debriefed until after all data were collected.

#### Experimental Feigning Conditions

The instructional sets that participants in the experimental feigning (i.e., factitious and malingering) conditions received, started by explaining to them that they were going to be asked to fill out a questionnaire and answer questions during two brief interviews and that they were to respond to all questions according to specific instructions that were detailed in the document they received. We refrained from directly instructing participants to act in a certain manner (e.g., feign a certain clinical presentation); rather, we prompted them to imagine themselves being in a certain setting and situation under particular circumstances with a specific background (which induced motives to feign symptomatology) and act as if they were in that situation and as if the described circumstances and background scenario were true.

Similar to Rogers et al. (2005), we employed the same setting and situation across all experimental conditions, thus controlling for “patient-role” effects (e.g., see Kroger & Turnbull, 1975). The setting that we used was the psychological support unit of Maastricht University, and the situation was an assessment by a student counselor. The psychological support unit of Maastricht University consists of psychologists (“student counselors”) who are specialized in providing support to students with psychological problems. The circumstances and background scenario that we employed involved fictional information about (a) a prior meeting that the participant had with a student counselor; (b) the feelings that the participant subsequently had toward the counselor; (c) the participant’s progress as a student (i.e., number of courses passed/failed) and their eligibility to complete the current academic year; and (d) the potential merits of feigning psychological problems during the upcoming “session” with the student counselor.

The experimental factitious condition was modeled after Rogers et al.’s (2005) instructional set for a *dependency on staff* factitious presentation, which is based on Cunnien’s (1997) differentiation of *dependency* and *demandingness* as interpersonal motivations driving factitious disorders. The *dependency* motivation involves immoderate need for care and support; the *demandingness* motivation involves frustration and resentment for unmet treatment needs (Cunnien, 1997). Participants in the factitious condition were informed that (a) their previous meeting with the student counselor was an extremely pleasant and comforting experience; (b) they had come to quietly admire and feel sustained by the student counselor; (c) their progress as a student was fine (no courses failed) and they were eligible to complete the current academic year; and (d) the only way to be assigned to frequent sessions with the student counselor (which they hoped for) was to appear as if they were suffering from serious psychopathology.

The circumstances and background scenario of the illness anxiety condition paralleled that of the factitious condition, save that (a) the prior meeting with the student counselor was described as having been an ordinary session, which did not provoke specific feelings toward the counselor, and (b) sometime after seeing the counselor, the participant had developed increasing worries about their health and had grown fearful of incurring serious pathology. Contrary to the factitious and malingering conditions, which included the suggestion to feign symptomatology, the illness anxiety condition merely asked participants to behave in a manner so as to be taken serious by the student counselor.

In the experimental malingering condition, participants were not asked to suppose that the prior meeting with the student counselor had been particularly fulfilling and led them to long for frequent sessions with the counselor (the factitious condition) or to imagine that they developed pressing health concerns (the illness anxiety condition). Instead, participants were impelled to presume that they failed several exams and were therefore ineligible to complete the current academic year and that the only way to be allowed to partake in the resits of the failed exams in the current academic year (with privileged exam regulations) would be to feign serious psychopathology and convince the counselor that their academic failure was caused by psychopathology.

To stimulate participants to engage with the experimental material and subsequent tests, we offered them a small monetary incentive (a €7.50 gift voucher, equivalent to $8.90). In addition, we stressed the importance of the research by pointing to the consequences of improper classification of feigned and genuine symptomatology (i.e., wrongfully allocated resources in health care, compensation, and damage claims). Participants in the three experimental conditions were informed that the questions they were to answer constituted tests designed to detect feigned symptomatology, and they were challenged to appear as credible as possible while striving to obtain their hypothetical incentive.

#### Nonclinical Control Condition

The nonclinical control condition was analogous to the experimental conditions, save that the scenario omitted incentives to feign symptoms (i.e., no dependency on student counselor, no denial to complete the current study year) and the instructions requested participants to answer all questions honestly.

#### Clinical Control Group

In contrast to the other groups, the clinical control group did not receive a monetary incentive to participate and did not undergo any experimental manipulation procedures: Instead, the importance of the research was highlighted and participants were implored to be completely truthful in answering all questions. Participants for the clinical control group were recruited via staff of the outpatient units of Radix Forensic Psychiatric Hospital, who alerted patients to the study and brought interested patients into contact with the researchers.

### Results

Means and corresponding 95% confidence intervals and standard deviations of SDI and SIMS scale scores of each group are summarized in Table 1. The mean SIMS scores indicate that the effect of our experimental manipulations to induce feigned symptomatology was similar across groups and comparable to that of earlier studies (see van Impelen et al., 2014, Table 6). Six participants of the clinical control group produced a SIMS score above 16 (which calls the validity of reported symptoms into question). The data of these six participants may reflect real-world feigning and are therefore interesting to compare the data of the experimental groups with. To increase the statistical reliability of analyses with these data, we merged it with that of the clinical participants in study 2 (see below) who also scored > 16 on the SIMS (*n* = 5): Together, these participants (*n* = 11) were treated as a single group in statistical analyses (see study 2, Table 7). Mann-Whitney *U* tests indicated that neither gender nor race (Caucasian vs. non-Caucasian) had a statistically significant effect on SDI or SIMS scale scores in any of the groups (all *U*s > 11.4, all exact, two-tailed *p*s > .14). There was also no statistically significant relation between participant’s age and SDI or SIMS scale scores in any of the groups (all Kendall rank correlation coefficients between −.27 and .23, all *p*s > .11). The main trend that emerges from Table 1 foretells the results that are reported below: The feigning groups generated higher SDI and SIMS scores than the clinical control group, and the factitious group yielded higher SDI (but not SIMS) scores than the other feigning groups.

**Table 1:** Symptom presentations on the Symptom and Disposition Interview (SDI) and the Structured Inventory of Malingered Symptomatology (SIMS) in study 1: mean scores, corresponding [95% confidence intervals], and (standard deviations)

Scale (score range)	Experimental groups				Clinical control group^a^	
	Factitious	Illness anxiety	Malingering	Nonclinical control	(SIMS ≤ 16)	(SIMS > 16)
	*n* = 24	*n* = 24	*n* = 24	*n* = 24	*n* = 28	*n* = 6
SDI US (0–28)	13.5 [11.3, 15.6] (5.1)	10.9 [8.3, 13.5] (6.2)	9.6 [7.3, 11.9] (5.4)	2.8 [1.6, 3.9] (2.6)	3.1 [1.6, 4.6] (3.8)	7.2 [2.4, 11.9] (4.5)
SDI CS (0–14)	8.6 [7.5, 9.7] (2.6)	8.0 [6.9, 9.2] (2.7)	7.7 [6.6, 8.7] (2.4)	3.0 [2.4, 3.7] (1.5)	4.8 [3.7, 6.0] (3.0)	8.8 [5.1, 12.6] (3.5)
SDI SR (0–28)	20.6 [18.9, 22.4] (4.1)	19.0 [17.2, 20.9] (4.5)	16.5 [14.7, 18.2] (4.2)	9.3 [7.4, 11.2] (4.5)	11.3 [9.6, 13.1] (4.5)	16.0 [11.7, 20.4] (4.1)
SDI IA (0–14)	12.0 [10.9, 13.0] (2.4)	7.4 [6.5, 8.3] (2.1)	7.5 [6.3, 8.8] (3.0)	4.7 [3.9, 5.5] (1.9)	4.4 [3.4, 5.4] (2.6)	6.3 [2.9, 9.8] (3.3)
SDI Total (0–84)	54.6 [50.1, 59.2] (10.8)	45.3 [39.9, 50.8] (13.0)	41.2 [36.6, 45.9] (11.0)	19.8 [16.5, 23.1] (7.9)	23.6 [19.6, 27.7] (10.4)	38.3 [24.2, 52.5] (13.5)
SIMS AF-N (−15–15)	0.7 [−0.9, 2.3] (3.9)	4.9 [3.5, 6.4] (3.4)	4.3 [2.7, 6.0] (3.9)	2.4 [1.7, 3.1] (1.7)	2.8 [1.9, 3.7] (2.3)	2.7 [−0.7, 6.0] (3.2)
SIMS Total (0–75)	23.2 [19.7, 26.7] (8.3)	23.5 [18.6, 28.3] (11.5)	21.5 [16.3, 26.7] (12.2)	4.8 [3.3, 6.2] (3.4)	9.0 [7.7, 10.4] (3.5)	23.3 [14.6, 32.0] (8.3)

*CS* common symptoms, *IA* illness anxiety, *SDI* Symptom and Disposition Interview, *SIMS* Structured Inventory of Malingered Symptomatology, *SR* sick role, *US* unlikely symptoms

^a^The group of participants with a SIMS score > 16 (*n* = 6) were excluded from all analyses involving the clinical control group; instead, the data of this group was combined with that of the participants with a SIMS score > 16 in study 2 to form a supplemental group for comparison with the experimental groups (see Table 7)

Relevant comparisons between SDI and SIMS scale scores of each group were examined through Cohen’s *d* effect sizes. The results are shown in Table 2 and reveal several important findings. First, all SDI scales demonstrated large statistically significant group differences between the feigning groups (factitious, illness anxiety, and malingering) and the clinical control group (*d*s ranging from 1.1 to 3.0, all *p*s < .001). Second, the sensitivity to feigned symptomatology of the SDI Total scale compares favorably with that of the SIMS (SDI *d*s, 1.6–2.9 vs. SIMS *d*s, 1.4–2.3). Third, the SDI SR, IA (Illness Anxiety), and Total scales distinguished well between the factitious and the malingering condition (*d*s 1.0 to 1.7, *p*s < .01). Fourth, in contrast to the SDI Total scale, the SIMS Total scale did not differentiate the factitious from the malingering condition (*d* = 0.2, *p* > .10). Fifth, no SDI scale evinced a statistically significant difference between the clinical and the nonclinical control group (*d*s −0.1 to 0.7, *p*s > .01), which indicates that genuine symptomatology does not have an undue effect on the other SDI scales, also in comparison with the effect that genuine pathology had on the SIMS Total scale (*d* = 1.2, *p* < .001).

**Table 2:** Differences in symptom presentation on the Symptom and Disposition Interview (SDI) and Structured Inventory of Malingered Symptomatology (SIMS) in study 1: Cohen’s *d* effect sizes and corresponding [95% confidence intervals]

Scale	Feigning groups vs. clinical control group			Feigning groups vs. each other			
	Factitious vs. clinical control	Illness anxiety vs. clinical control	Malingering vs. clinical control	Factitious vs. malingering	Illness anxiety vs. malingering	Factitious vs. illness anxiety	Clinical control vs. nonclinical control
SDI US	2.3** [1.6, 3.0]	1.5** [0.9, 2.1]	1.4** [0.8, 2.0]	0.7 [0.1, 1.3]	0.2 [−0.4, 0.8]	0.5 [−0.1, 1.1]	0.1 [−0.4, 0.6]
SDI CS	1.3** [0.7, 1.9]	1.1** [0.5, 1.7]	1.1** [0.5, 1.7]	0.4 [−0.2, 1.0]	0.1 [−0.5, 0.7]	0.2 [−0.4, 0.8]	0.7 [0.1, 1.3]
SDI SR	2.2** [1.5, 2.9]	1.7** [1.1, 2.3]	1.2** [0.6, 1.8]	1.0* [0.4, 1.6]	0.6 [0.0, 1.2]	0.4 [−0.2, 1.0]	0.4 [−0.2, 0.9]
SDI IA	3.0** [2.2, 3.8]	1.3** [0.7, 1.9]	1.1** [0.5, 1.7]	1.7** [1.0, 2.4]	0.0 [−0.6, 0.6]	2.0** [1.3, 2.7]	−0.1 [−0.6, 0.4]
SDI Total	2.9** [2.1, 3.7]	1.9** [1.2, 2.6]	1.6** [1.0, 2.2]	1.2** [0.6, 1.8]	0.3 [−0.3, 0.9]	0.8 [0.2, 1.4]	0.4 [−0.2, 0.9]
SIMS AF-N	−0.7 [−1.3, −0.1]	0.7 [0.1, 1.3]	0.5 [−0.1, 1.1]	−0.9* [−1.5, −0.3]	0.2 [−0.4, 0.8]	−1.1* [−1.7, −0.5]	0.2 [−0.3, 0.7]
SIMS Total	2.3** [1.6, 3.0]	1.8** [1.1, 2.4]	1.4** [0.8, 2.0]	0.2 [−0.4, 0.8]	0.2 [−0.4, 0.8]	0.0 [−0.6, 0.6]	1.2** [0.6, 1.8]

*CS* common symptoms, *IA* illness anxiety, *SDI* Symptom and Disposition Interview, *SIMS* Structured Inventory of Malingered Symptomatology, *SR* sick role, *US* unlikely symptoms

*Cohen’s *d* is significant (i.e., > 0.0) at the *p* < .01 level

**Cohen’s *d* is significant (i.e., > 0.0) at the *p* < .001 level

Whereas the above findings are all confirmatory with regard to our hypotheses, two additional findings were not so: The first is that we expected the SDI, and its IA scale, in particular, to discriminate between the illness anxiety condition and the malingering condition, but this did not materialize (*d*s 0.0–0.6, *p*s > .01). The second is that the experimental factitious group scored significantly lower on the SIMS AF-N index than the malingering group (*d* = −0.9, *p* < .01), which is opposite to what Rogers et al. (2005) found.

Table 3 displays area under the curve (AUC) values, which are a measure of overall diagnostic accuracy, with values from .50 to .70 signifying low accuracy, .70 to .90 moderate accuracy, and > .90 high accuracy (Swets, 1988). The SDI scales, like the SIMS, are moderately to highly effective in correctly classifying feigned and authentic symptom presentations (AUCs .75–.97, *p*s < .01), with the Total scale appearing marginally superior over the subscales. Despite being dedicated to detecting feigned symptomatology, the US scale is not significantly more accurate than the SR or IA scales in this respect, and the CS scale lags slightly behind.

**Table 3:** Diagnostic utility of the Symptom and Disposition Interview (SDI) and the Structured Inventory of Malingered Symptomatology (SIMS) in study 1: area’s under the receiver operating characteristic curve and corresponding [95% confidence intervals]

Scale	Feigning groups vs. clinical control group			Feigning groups vs. each other		
	Factitious vs. clinical control	Illness anxiety vs. clinical control	Malingering vs. clinical control	Factitious vs. malingering	Illness anxiety vs. malingering	Factitious vs. illness anxiety
SDI US	.94** [.88, 1.00]	.88** [.78, .97]	.85** [.74, .95]	.69 [.54, .84]	.55 [.38, .72]	.62 [.46, .79]
SDI CS	.83** [.71, .94]	.77* [.65, .90]	.75* [.62, .88]	.62 [.46, .78]	.53 [.36, .69]	.58 [.41, .74]
SDI SR	.93** [.87, 1.00]	.88** [.79, .97]	.80** [.68, .92]	.76* [.63, .90]	.67 [.51, .82]	.59 [.43, .75]
SDI IA	.97** [.90, 1.00]	.81** [.70, .93]	.79** [.66, .91]	.88** [.76, .99]	.50 [.33, .66]	.93** [.85, 1.00]
SDI Total	.97** [.93, 1.00]	.91** [.82, .99]	.88** [.79, .97]	.82** [.70, .94]	.58 [.41, .74]	.71 [.56, .86]
SIMS AF-N	.32 [.16, .47]	.70 [.55, .84]	.66 [.50, .82]	.25* [.11, .39]	.52 [.35, .69]	.21* [.08, .34]
SIMS Total	.95** [.88, 1.00]	.89** [.79, .99]	.83** [.70, .95]	.54 [.37, .71]	.55 [.38, .71]	.52 [.35, .68]

*CS* common symptoms, *IA* illness anxiety, *SDI* Symptom and Disposition Interview, *SIMS* Structured Inventory of Malingered Symptomatology, *SR* sick role, *US* unlikely symptoms

*Area under the curve is significantly greater than .5 (i.e., above chance level) at the *p* < .01 level

**Area under the curve is significantly greater than .5 (i.e., above chance level) at the *p* < .001 level

The SDI Total scale and the IA scale, especially, are fairly accurate in differentiating participants in the factitious conditions from those in the malingering condition (AUCs .82 and .88, *p*s < .01); the SR scale is less accurate, but still useful (AUC = .76, *p* < .01). No scale discriminated between analogue illness anxiety and malingered symptomatology (AUCs .50–.67, *p*s > .01).

In line with our hypotheses, the SIMS performed at chance level when employed to tell participants in the factitious condition apart from those in the malingering condition (AUC .54, *p* > .10). Although the SIMS AF-N index displayed a discriminatory ability between the factitious and the malingering condition, this efficacy was not in the expected direction; instead of performing above chance level, the AF-N index performed significantly below chance in classifying factitious and malingering participants (AUC .25, *p* < .01).

## Study 2

Past studies found the results of analogue feigning research to be dependent on the context and relevance of the scenario that is used as experimental manipulation (Merckelbach, Smeets, & Jelicic, 2009; Rogers & Cruise, 1998). In light of the encouraging outcomes of study 1, we decided to test to what extent the results would hold in a civil vs. a criminal law scenario, both of which are less familiar and relevant to participants than the “student counselor” scenarios used in study 1. Factitious symptom presentations were instilled through a civil law scenario, and malingered presentations were elicited via a civil and a criminal law scenario, respectively (see below).

In study 1, we provided participants in the experimental conditions with instructional sets 2 days before the test session would take place. For study 2, we handed the instructional sets to participant at the start of the test session and allowed them only 20 min preparation time (with access to the Internet) before the study materials were administered. The reason for this modification is twofold: First, we wanted to align this part of the procedure more with previous studies, such as that by Rogers et al. (2005), and second, we wanted less variation in preparation time between participants, as some participants in study 1 took considerable time to prepare while others did not take any time at all. Because real-world feigners typically have ample time to prepare for feigning, a short preparation time was retained to preserve some ecological validity in this regard.

Three participants in the experimental conditions of study 1 produced very low SIMS scores (i.e., < 5, which is lower than clinical controls and comparable to nonclinical controls; see Table 1) despite passing all manipulation checks (i.e., they correctly recalled the scenario and instructions, and endorsed compliance). This led us to realize that study 1 lacked a fundamental manipulation check; a standard clinical measure of symptomatology with norm-referenced cut scores. We avoided this shortcoming in study 2 by including the *Brief Symptom Inventory* (BSI; Derogatis & Melisaratos, 1983) to check whether participants in the experimental conditions presented with clinically relevant symptomatology.

The addition of the BSI and the 20-min preparation prolonged the total session time with roughly 30 min. Therefore, we raised the value of the gift voucher that participants received for participating in one of the experimental conditions from €7.50 to €12.50 (equivalent to $14.90).

Another change is the elimination of the nonclinical control group: Although the scores of such a group are useful to quantify the sensitivity of an SVT to authentic symptomatology, they are not helpful in distinguishing authentic from feigned symptomatology. Akin to study 1, we contrasted the scores of the experimental groups with those of a clinical control group in which SIMS scores above 16 served as exclusion criterion.

Aside from the changes just mentioned, study 2 is an emulation of study 1. Therefore, our general hypotheses remained the same; we predicted the SDI to approach the SIMS in classifying feigned presentations and exceed the SIMS in differentiating between factitious and malingered presentations. Based on Merckelbach et al. (2009), we expected the civil law scenario to prompt less perceptible feigning than the criminal law scenario.

### Method

The methods of study 2 mirrored those of study 1, save for the modifications outlined above, which include new experimental materials (civil and criminal law scenarios), 20 min preparation time during the session instead of two full days leading up to the session, inclusion of a measure of genuine symptomatology as manipulation check for the experimental conditions, and the exclusion of a nonclinical control group.

### Participants

In addition to graduate and undergraduate psychology students at Maastricht University, members of the general population were recruited as participants for the three experimental conditions (*n* = 32 per condition). Only native Dutch speakers who did not partake in study 1 were eligible for participation. None of the participants reported a history of severe mental illness. Eight participants were excluded from the statistical analyses because they did not simulate a significant level of symptomatology (see below). The remaining sample for the experimental conditions consisted of 64 students and 24 general population members (approximately evenly distributed over the three experimental conditions). The final sample for the experimental conditions (*n* = 88) was predominantly female (74%; *n* = 65) and Caucasian (93%; *n* = 82), with a mean age of 27.4 years (*SD* = 12.5; range 18–65).

For the clinical control condition, participants (*n* = 40) were recruited at an inpatient unit (*n* = 25) and an outpatient unit (*n* = 15) of the same forensic psychiatric hospital that provided the clinical sample for study 1; Radix Forensic Psychiatric Hospital (see the method section of study 1). All but one participant were male (98%; *n* = 39). The majority was Caucasian (83%; *n* = 33). The mean age was 36.5 years (*SD* = 9.2; range 21–61 years). Offenses for which participants were convicted often involved drugs, violence, theft, or sexual abuse. The most prevalent forms of psychopathology were substance disorders (60%) and personality disorders (60%), which occurred frequently with comorbid symptomatology, including schizophrenia, autism, depression, intellectual disability, or posttraumatic stress disorder.

### Measures

Identical to study 1, the SDI, the SIMS, and the M-FAST were administered to participants; only the SDI and the SIMS are included in the present analyses.

#### Brief Symptom Inventory

The *Brief Symptom Inventory* (BSI; Derogatis & Melisaratos, 1983) is a 53-item self-report scale of psychopathology. It comprises nine subscales that represent various psychopathological domains, ranging from affective and anxiety disorders to cognitive impairments and somatic symptoms. Each item consists of a symptom description and a 5-point Likert-type scale (range 0–4) that respondents use to indicate to what extent they felt distressed by the symptom during the past week. BSI Total and scale scores are computed by adding appropriate item scores and dividing the result by the number of items (i.e., scale scores are the means of their item scores). We employed the Dutch translation and norms constructed by De Beurs and Zitman (2006; Cronbach’s α = .96). To check whether participants in the experimental feigning conditions simulated clinically relevant levels of symptomatology, we used a Total (i.e., mean item) score of 0.50 as lower bound, which is associated with a sensitivity of .84 and a specificity of .70 (De Beurs & Zitman, 2006).

### Procedure

The procedures of study 2 were equivalent to those of study 1, except for the changes discussed earlier: We included the BSI in the test battery; we increased the compensation that participants in the experimental conditions received from €7.50 to €12.50 which is roughly $14.90; and instead of sending the instructional sets to participants 2 days before their test session, we presented the instructional sets at the beginning of the test session and gave participants 20 min preparation time (with access to the Internet) after they studied the instructional materials.

#### Experimental Feigning Conditions

The instructional sets of study 2 followed the same approach that was employed in study 1: Participants were prompted to act as if they were in a certain setting, in a specific situation, under particular circumstances with a given background. We used the same hypothetical setting and situation in the three experimental conditions; all participants were asked to imagine that they were being evaluated by a court-ordered psychologist at a forensic clinic. The fictional circumstances and background of the psychological evaluation were similar across conditions, but varied in important aspects. Participants read a detailed description of how they had been provoked into a mildly violent altercation at a college party that they had attended 3 weeks prior. The outcome of the incident varied across conditions and served to incentivize factitious or malingered symptom presentations, as detailed below.

In the experimental factitious condition, the outcome of the incident was that the participant sustained superficial injuries and started a civil lawsuit against their opponent. In the process of the lawsuit, which ended up being dismissed, the participant got into contact with a psychologist whom they soon came to revere and feel dependent on and were longing to keep into contact with; the only way to get into treatment and keep seeing the psychologist was to appear as if suffering from serious symptomatology. The *dependency on staff* factitious presentation as formulated by Cunnien (1997) and interpreted by Rogers et al. (2005) served again as prototype of the interpersonal motivations behind factitious disorders.

The experimental civil law malingering condition featured the same outcome of the college party incident; participants were asked to suppose that they suffered only minor injuries but still instigated a civil lawsuit. However, instead of being informed that the lawsuit was unsuccessful, participants were informed that the outcome of the lawsuit depended on the evaluation by the psychologist and that presenting as if suffering from serious symptomatology would lead to substantial damages being awarded to them.

In the criminal law malingering condition, the college party altercation resulted in severe (authentic) injuries for the adversary and an ensuing criminal trial for the participant. Analogous to the civil law malingering condition, the outcome of the trial depended on the evaluation by the psychologist, with a false presentation of serious symptomatology being the only way to a avoid criminal responsibility and corresponding punishment.

Other than the increased monetary incentive (€12.50 gift voucher instead of €7.50), we did not change our methods to encourage participants to engage with and act according to the experimental materials: Thus, we emphasized the importance of the research and challenged participants to appear credible on the tests while attempting to obtain their hypothetical incentive.

#### Clinical Control Group

The recruitment and test session procedures for the clinical control group were identical to those of study 1: Participants were recruited via personnel of the patient units of Radix Forensic Psychiatric Hospital, and were only requested to be completely honest during the test session. Participants’ engagement and effort was not stimulated by any financial incentives, but by underscoring the importance of the research.

### Results

Eight participants (two in the factitious condition, three in the civil law malingering condition, and three in the criminal law malingering condition) were excluded from the analyses because their BSI score was below 0.50, which suggests that they did not simulate a clinically relevant level of symptomatology. The remaining participants in the feigning conditions typically presented as patients on the BSI, generating mean scores between 1.4 and 2.0, which is slightly higher than the mean score of the heterogeneous clinical group (N = 992) of De Beurs and Zitman (2006; *M* = 1.2). The mean SIMS scores of the experimental feigning groups are marginally lower than those of the feigning groups of study 1, yet still similar to those of previous studies (van Impelen et al., 2014). Five participants of the clinical control group scored above 16 on the SIMS. These five participants were pooled with the clinical participants in study 1 who also scored > 16 on the SIMS (*n* = 6): Together, these participants were handled as a single group (*n* = 11) for comparison with the factitious conditions of studies 1 and 2 (see Table 7). Mann-Whitney *U* tests showed that neither gender nor race (Caucasian vs. non-Caucasian) had a statistically significant effect on SDI or SIMS scale scores in any of the groups (all *U*s > 8.4, all exact, two-tailed *p*s > .09). Likewise, age was not statistically related to SDI or SIMS scale scores in any of the groups (all Kendall rank correlation coefficients between −.25 and .24, all *p*s > .16).

Table 4 displays group means and corresponding 95% confidence intervals and standard deviations of SDI and SIMS scale scores. The pattern that can be observed is comparable to that in study 1: The experimental feigning groups produced higher SDI and SIMS scores than the clinical control group, and the factitious group scored higher—albeit to a lesser extent—on the SDI, but not on the SIMS, than the other feigning groups.

**Table 4:** Symptom presentations on the Symptom and Disposition Interview (SDI) and the Structured Inventory of Malingered Symptomatology (SIMS) in study 2: mean scores, corresponding [95% confidence intervals], and (standard deviations)

Scale (score range)	Experimental groups			Clinical control group^a^	
	Factitious (civil law) *n* = 30	Malingering (civil law) *n* = 29	Malingering (criminal law) *n* = 29	(SIMS ≤ 16) *n* = 35	(SIMS > 16) *n* = 5
SDI US (0–28)	13.0 [11.3, 14.7] (4.5)	11.1 [9.3, 12.9] (4.7)	12.4 [10.6, 14.2] (4.7)	3.7 [2.1, 5.2] (4.4)	11.4 [6.9, 15.9] (3.6)
SDI CS (0–14)	7.0 [6.3, 7.8] (2.0)	6.0 [5.1, 6.8] (2.2)	6.8 [6.0, 7.6] (2.1)	4.5 [3.7, 5.4] (2.5)	8.2 [5.0, 11.4] (2.6)
SDI SR (0–28)	17.0 [15.5, 18.5] (4.0)	14.8 [13.0, 16.5] (4.5)	14.4 [12.6, 16.2] (4.8)	11.8 [10.1, 13.6] (5.1)	15.2 [10.8, 19.6] (3.6)
SDI IA (0–14)	6.0 [5.2, 6.8] (2.2)	6.3 [5.5, 7.1] (2.1)	4.0 [3.3, 4.7] (1.8)	4.7 [3.9, 5.6] (2.3)	6.2 [3.5, 8.9] (2.2)
SDI Total (0–84)	43.0 [39.7, 46.3] (8.7)	38.1 [33.9, 42.3] (11.0)	37.6 [33.9, 41.2] (9.7)	24.7 [20.8, 28.6] (11.4)	41.0 [30.1, 51.9] (8.7)
SIMS AF-N (−15–15)	3.3 [2.3, 4.2] (2.5)	2.5 [1.5, 3.5] (2.6)	4.1 [3.0, 5.2] (2.9)	3.2 [2.5, 4.0] (2.2)	2.8 [0.4, 5.2] (1.9)
SIMS Total (0–75)	20.4 [16.8, 24.1] (9.7)	16.8 [12.2, 21.4] (12.1)	20.1 [16.6, 23.6] (9.2)	7.2 [5.9, 8.5] (3.8)	21.0 [15.7, 26.3] (4.2)
BSI Total (0.0–4.0)	2.0 [1.8, 2.2] (0.5)	1.4 [1.1, 1.6] (0.6)	1.7 [1.5, 1.9] (0.5)	NA	NA

*CS* common symptoms, *IA* illness anxiety, *SDI* Symptom and Disposition Interview, *SIMS* Structured Inventory of Malingered Symptomatology, *SR* sick role, *US* unlikely symptoms

^a^The group of participants with a SIMS score > 16 (*n* = 5) were excluded from all analyses involving the clinical control group; instead, the data of this group was combined with that of the participants with a SIMS score > 16 in study 1 to form a supplemental group for comparison with the experimental groups (see Table 7)

Differences in scores on SDI and SIMS scales between the various groups were once more quantified through Cohen’s *d* effect sizes. The results are on display in Table 5 and corroborate many, but not all, findings of study 1: While the SDI US and Total scales again performed in the same league as the SIMS by manifesting large group differences between the feigning groups (factitious, civil law malingering, and criminal law malingering) and the clinical control group (*d*s in the range of 1.2 to 2.1, *p*s < .001), the other SDI scales were less effective in this regard.

**Table 5:** Differences in symptom presentation on the Symptom and Disposition Interview (SDI) and Structured Inventory of Malingered Symptomatology (SIMS) in study 2: Cohen’s *d* effect sizes and corresponding [95% confidence intervals]

Scale	Feigning groups vs. clinical control group			Factitious group vs. other feigning groups	
	Factitious (civil) vs. clinical control	Malingering (civil) vs. clinical control	Malingering (criminal) vs. clinical control	Factitious (civil) vs. malingering (civil)	Factitious (civil) vs. malingering (criminal)
SDI US	2.1** [1.5, 2.7]	1.6** [1.0, 2.2]	1.9** [1.3, 2.5]	0.4 [−0.1, 0.9]	0.1 [−0.4, 0.6]
SDI CS	1.1** [0.6, 1.6]	0.6 [0.1, 1.1]	1.0** [0.5, 1.5]	0.5 [0.0, 1.0]	0.1 [−0.4, 0.6]
SDI SR	1.1** [0.6, 1.6]	0.6 [0.1, 1.1]	0.5 [0.0, 1.0]	0.5 [0.0, 1.0]	0.6 [0.1, 1.1]
SDI IA	0.6 [0.1, 1.1]	0.7* [0.2, 1.2]	−0.3 [−0.8, 0.2]	−0.1 [−0.6, 0.4]	1.0 [0.5, 1.5]
SDI Total	1.8** [1.2, 2.4]	1.2** [0.7, 1.7]	1.2** [0.7, 1.7]	0.5 [0.0, 1.0]	0.6 [0.1, 1.1]
SIMS AF-N	0.0 [−0.5, 0.5]	−0.3 [−0.8, 0.2]	0.4 [−0.1, 0.9]	0.3 [−2, 0.8]	−0.3 [−0.8, 0.2]
SIMS Total	1.8** [1.2, 2.4]	1.1** [0.6, 1.6]	1.9** [1.3, 2.5]	0.3 [−0.2, 0.8]	0.0 [−0.5, 0.5]

*CS* common symptoms, *IA* illness anxiety, *SDI* Symptom and Disposition Interview, *SIMS* Structured Inventory of Malingered Symptomatology, *SR* sick role, *US* unlikely symptoms

*Cohen’s *d* is significant (i.e., > 0.0) at the *p* < .01 level

**Cohen’s *d* is significant (i.e., > 0.0) at the *p* < .001 level

In contrast to study 1, the SDI failed to distinguish the factitious group from the malingering groups (*d*s − 0.1–0.6, *p*s > .01). The potential that the SIMS AF-N index showed in the factitious vs. malingering comparisons of study 1 (*d* = −0.9, but cf. *d*s 1.1–1.4 of Rogers et al., 2005) did not replicate in study 2, where the AF-N index was as ineffective as the SIMS Total scale (*d*s − 0.3–0.3, *p*s > .10).

The diagnostic accuracy of the SDI (the US as well as the Total scale) and the SIMS for detecting feigned symptomatology, as represented by AUC values in Table 6, is slightly lower than in study 1, yet still acceptable, with the SDI US scale now even performing on a par with the SDI and SIMS Total scales (AUCs; SDI US; .90–.92, SDI Total; .81–.90, SIMS Total; .78–.92, all *p*s, < .001). The efficacy of the other SDI scales (CS, SR, and IA) in classifying feigned symptomatology fluctuated between poor and moderate (AUCs .43–.79, *p*s > .10–< .001).

**Table 6:** Diagnostic utility of the Symptom and Disposition Interview (SDI) and the Structured Inventory of Malingered Symptomatology (SIMS) in study 2: area’s under the receiver operating characteristic curve and corresponding [95% confidence intervals]

Scale	Feigning groups vs. clinical control group			Factitious group vs. other feigning groups	
	Factitious (civil) vs. clinical control	Malingering (civil) vs. clinical control	Malingering (criminal) vs. clinical control	Factitious (civil) vs. malingering (civil)	Factitious (civil) vs. malingering (criminal)
SDI US	.92** [85., 1.00]	.90** [.81, .98]	.91** [.84, .99]	.64 [.49, .78]	.54 [.39, .69]
SDI CS	.79** [.68, .90]	.67 [.54, .80]	.76** [.65, .88]	.63 [.49, .77]	.51 [.36, .66]
SDI SR	.78** [.67, .89]	.66 [.52, .79]	.65 [.52, .79]	.65 [.51, .79]	.66 [.52, .80]
SDI IA	.67 [.54, .80]	.70* [.58, .83]	.43 [.29, .57]	.47 [.32, .62]	.75* [.62, .87]
SDI Total	.90** [.82, .98]	.81** [.70, .92]	.82** [.71, .92]	.63 [.49, .78]	.67 [.53, .81]
SIMS AF-N	.50 [.36, .64]	.44 [.30, .58]	.60 [.46, .75]	.57 [.42, .72]	.41 [.26, .56]
SIMS Total	.90** [.83, .98]	.78** [.66, .90]	.92** [.85, .98]	.63 [.49, .77]	.51 [.37, .66]

*CS* common symptoms, *IA* illness anxiety, *SDI* Symptom and Disposition Interview, *SIMS* Structured Inventory of Malingered Symptomatology, *SR* sick role, *US* unlikely symptoms

*Area under the curve is significantly greater than .5 (i.e., above chance level) at the *p* < .01 level

**Area under the curve is significantly greater than .5 (i.e., above chance level) at the *p* < .001 level

In study 1, the SDI US and SR, and particularly the IA and Total scales, achieved significant accuracy in differentiating participants in the factitious condition from those in the malingering condition (AUCs .69–.88). The results of study 2 are not corroborative: No scale was effective in differentiating the factitious condition from the civil malingering condition (AUCs = .47–.65, *p*s > .01), and only the IA scale managed to discriminate the factitious from the criminal malingering condition (AUC = .75, *p* < .01).

As in study 1, the SIMS Total scale did not demonstrate any precision in separating factitious from malingered experimental conditions. The promise that the SIMS AF-N index showed in study 1—AUC .25, which translates to .75 if lower AF-N index scores are taken to be indicative of the factitious condition, instead of higher scores as found by Rogers et al., 2005—did not transpire in study 2; AUCs were .41–.57, *p*s > .10.

Several participants in the clinical control groups of study 1 and study 2 scored above 16 on the SIMS, which compromises the validity of their symptom report and may reflect real-world feigning. Because these groups were recruited at the same forensic psychiatric hospital (at different points in time), received identical instructions (i.e., to respond honestly to all questions), and produced similar SIMS Total scores (means 23.3 vs. 21.0; *t*(9) = 0.57, *p* = .58), we grouped these participants together (*n* = 11) and employed them as an additional feigning group to compare the experimental factitious groups with. The results are on display in Table 7 and concur with the results from the experimental malingering-factitious comparisons: The SDI SR and Total scale, and above all the IA scale, distinguished well between the clinical group that produced SIMS scores > 16 and the factitious group of study 1 (*d*s 1.3 to 2.3, AUCs .83–.95, *p*s < .01), but not the factitious group of study 2 (*d*s −0.1 to 0.4, AUCs .46–.59, *p*s > .10). The SIMS AF-N index did not attain accuracy above chance level in discriminating the clinical SIMS > 16 group from the factitious group of either study.

**Table 7:** Comparison of the factitious groups of study 1 and study 2 with the clinical groups of study 1 and study 2 who produced SIMS scores > 16

Scale	Mean score [95% CI] (SD)	Cohen’s *d* effect sizes [95% confidence intervals]		Area’s under the receiver operating characteristic curve [95% confidence intervals]	
	Clinical (SIMS > 16)^a^ *n* = 11	Factitious study 1 vs. clinical SIMS > 16	Factitious study 2 vs. clinical SIMS > 16	Factitious study 1 vs. clinical SIMS > 16	Factitious study 2 vs. clinical SIMS > 16
SDI US	9.1 [6.1, 12.1] 4.5	0.9 [0.1, 1.6]	0.9 [0.2, 1.6]	.74 [.57, .91]	.73 [.56, .90]
SDI CS	8.6 [6.5, 10.6] 3.0	0.0 [−0.7, 0.7]	−0.7 [−1.4, 0.0]	.53 [.31, .74]	.34 [.13, .56]
SDI SR	15.6 [13.1, 18.1] 3.7	1.3* [0.5, 2.1]	0.4 [−0.3, 1.1]	.83* [.70, .96]	.59 [.41, .78]
SDI IA	6.3 [4.5, 8.1] 2.7	2.3** [1.4, 3.2]	−0.1 [−0.8, 0.6]	.95** [.87, 1.00]	.46 [.24, .68]
SDI Total	39.6 [32.1, 47.0] 11.1	1.4* [0.6, 2.2]	0.4 [−0.3, 1.1]	.86* [.75, .98]	.57 [.36, .78]
SIMS AF-N	2.7 [1.0, 4.5] 2.6	−0.6 [−1.3, 0.1]	0.2 [−0.5, 0.9]	.31 [.14, .49]	.57 [.38, .77]
SIMS Total	22.3 [17.9, 26.7] 6.6	0.1 [−0.6, 0.8]	−0.2 [−0.9, 0.5]	.55 [.35, .75]	.43 [.25, .60]

*CS* common symptoms, *IA* illness anxiety, *SDI* Symptom and Disposition Interview, *SIMS* Structured Inventory of Malingered Symptomatology, *SR* sick role, *US* unlikely symptoms

*Cohen’s d is significant (i.e., > 0.0) at the p < .01 level / Area under the curve is significantly greater than .5 (i.e., above chance level) at the *p* < .01 level

**Cohen’s d is significant (i.e., > 0.0) at the p < .001 level / Area under the curve is significantly greater than .5 (i.e., above chance level) at the *p* < .001 level

^a^This group consists of participants with a SIMS score > 16 in the clinical control groups of study 1 (*n* = 6) and study 2 (*n* = 5)

We were also interested in the reliability of the SDI scales and, therefore, we computed Cronbach’s alpha values over the pooled data of studies 1 and 2 (*N* = 258). The reliability of the Total scale was high (*α* = .91), closely followed by that of the US scale (*α* = .86); the alpha values of the other scales were acceptable (CS *α* = .70, SR *α* = .71, IA *α* = .74).

## Discussion

The present studies address an issue that is often overlooked in clinical practice and practically neglected in research: The structured assessment of internal incentives for feigned symptom presentations. We took a psychometric approach to this issue and developed the *Symptom and Disposition Interview* (SDI), which screens for feigned symptomatology *and* assesses potential internal incentives for feigning (i.e., the need to assume the sick role). The SDI consists of four scales: Unlikely Symptoms (14 items measuring noncredible symptom reporting), Common Symptoms (7 items serving to disguise the symptom validity aspect of the Unlikely Symptom scale and lower its sensitivity to authentic symptomatology), Sick Role (14 items querying the readiness to engage in patient-related activities), and Illness Anxiety (7 items covering sensitivity to somatic symptoms and distress over potential pathology, which we included because the internal motivations that drive factitious symptom presentations are similar to those that underlie somatic symptomatology). To investigate the potential merits of the SDI, we compared it to a traditional symptom validity test (the Structured Inventory of Malingered Symptomatology, SIMS; Smith & Burger, 1997) in two analogue studies, each with factitious and malingering conditions (*n* = 24–30 per condition) and a clinical control group (*n* = 34, *n* = 40). The first and foremost point that can be taken from our two studies is that the assessment of internal incentives can be incorporated into a symptom validity test without sacrificing efficacy in detecting feigned symptomatology, as the SDI achieved parity with the SIMS in this respect.

The second conclusion that can be drawn is that the utility of the SDI in differentiating experimental factitious symptom presentations from malingered symptom presentations is sufficiently promising to justify future research: The diagnostic accuracy (i.e., AUC) of the SR scale varied between .65 and .80. In designing the SR scale, we assembled items that diverge widely with regard to their topic and approach, yet all approaches were taken to gauge the strength of an internal motive to feign symptomatology. In this light, the reliability of the SR scale (14 items, α = .71) is satisfactory.

Nonetheless, the assessment of factitious symptom presentations can be improved by advancing from rationally derived approaches to empirically established detection strategies. In this regard, future research may revolve around self-reported illness anxiety and somatic sensitivity. The rationale behind the development of the IA scale was that any measure of somatoform symptomatology would indirectly mark internal incentives for such symptomatology as well as for factitious symptomatology, because the incentives for both symptomatologies are thought to be similar (the main difference being that those of somatoform symptomatology largely reside outside awareness; see Krahn et al., 2008). The items of the IA scale represent two potential detection-based strategies: The first encompasses sensitivity to, and frequency of, mild somatic symptomatology, and the second involves distress and anxiety over potential illnesses and health risks. More research on these strategies seems required.

The logical next step toward structured assessment of factitious symptom presentations is known-groups research (Rogers, 2008a). Rather than theorizing as to why some items or strategies work well and others do not in analogue research and attempting to gain more insight through additional analogue studies, it is pertinent to put positive findings in analogue research to the test in clinical group comparisons (i.e., patients with factitious disorder vs. real-world malingerers). For example, finding out why items referring to somatic sensitivity and illness anxiety worked (particularly well in study 1) in differentiating experimental factitious symptomatology from experimental malingered symptomatology is of subsidiary importance to finding out what the value of such items is in forensic and clinical work. This is not to say that the theoretical foundation of a detection strategy is unimportant—in fact, it may well originate from there—but that the ultimate test of its adequacy is its utility in clinical practice (Meehl, 1945, but see Butcher, 2000).

The third outcome of the current studies is that the detection strategies described above did not consistently produce better results collectively than individually. For instance, the IA scale outperformed the Total scale in the majority of factitious vs. malingering comparisons (see Tables 3 and 6). Another example is the US scale attaining greater accuracy in discriminating feigned symptomatology from authentic symptomatology than the Total scale in study 2 (see Table 6).

The fourth point that can be deduced from the data is that the CS scale of the SDI, which is an auxiliary scale referring to different (credible) psychiatric symptoms, succeeds in its functions that were tested in the present studies, these being (a) lowering the sensitivity of the US and IA scales to genuine symptomatology—by providing the opportunity to endorse such symptomatology—and (b) supporting the detection of feigned symptomatology. As can be seen in Table 2, the effect of genuine symptomatology on CS scale scores is substantially greater than on US and IA scale scores (*d*s 0.7 vs. 0.1 and −0.1). Tables 3 and 6 show that the CS scale possesses respectable diagnostic efficacy in classifying feigned symptomatology (AUCs .67–.83). The latter result reflects the common finding that malingerers typically endorse a wider variety and higher severity of symptoms than do authentic patients (hence, the effectiveness of the *indiscriminant symptom endorsement* and *symptom severity* detection strategies; Rogers, 2008b).

The last conclusion that flows from the present data is that the SIMS AF-N index, which yielded encouraging results in Rogers et al. (2005), does not hold up, as it delivered conflicting results. Although the AF-N index had diagnostic value for the factitious vs. malingering comparison in study 1, this was because the factitious condition induced low AF-N scores, which is the inverse of what was reported by Rogers et al. (2005). In study 2, the AF-N index did not evidence any diagnostic utility. The most plausible explanation for the discrepant findings is one that Rogers et al. (2005) entertained: That the observed AF-N differences between factitious and malingering groups are artifacts of the instructional sets.

An important constraint of the current studies is that it is not possible to establish to what extent the analogue factitious symptom presentations resemble real factitious symptom presentations; the clinical presentation of analogue simulators may differ considerably from that of genuine patients with factitious disorder. To some degree, this limitation is inherent in analogue designs, but the lack of published data on the standardized assessment of factitious disorders compounds this issue for the present studies. The only suitable data available for comparison are those of Rogers et al. (2005). The mean SIMS scores obtained by participants in the factitious conditions of Rogers et al. (2005) were 15.1 and 16.8, which is notably lower than the 23.2 and 20.4 that participants in our factitious conditions obtained. These discrepancies may stem from the fact that the samples of Rogers et al. (2005) consisted exclusively of doctoral psychology students, whereas our samples also included undergraduate students. Future research that samples actual patients with factitious disorder will have to establish whether the doctoral students of Rogers et al. (2005) were spot on sophisticated or too subtle in their feigning: The mean SIMS scores of malingerers (23.7–38.2; van Impelen et al., 2014) suggest the latter to be more likely. However, these data only pertain to the feigned symptomatology part of factitious disorder and not to the motivational part (e.g., *dependency* or *demandingness*; see Cunnien, 1997), which is arguably more difficult to simulate. In addition to distilling the clinical characteristics of factitious symptom presentations, subsequent research in clinical populations may investigate whether the rationally derived detection strategies of the SDI represent well-defined dimensions (through, for example, factor analysis).

Another serious limitation of the present data is the unequal gender distribution. It may be considered tolerable that the samples for the experimental groups are predominantly female because the majority of patients with factitious disorder are also female (Bass & Halligan, 2014; Yates & Feldman, 2016), yet it is problematic that the samples for the clinical control groups are almost exclusively male. Although Mann-Whitney *U* tests indicate that gender did not have a significant effect on SDI or SIMS scores, the small numbers of males in the experimental groups and females in the clinical control groups may be fatal to the reliability of these analyses. Nevertheless, the results of the Mann-Whitney *U* tests square with previous research on the SIMS, which revealed that gender has no impact on SIMS scores (van Impelen et al., 2014). Other sample characteristics that impose considerable constraints are the restriction in age (mostly young adults in the experimental groups), race (nearly all participants were Caucasian, as were the researchers that administered the tests), and education (above average education in the experimental groups and below average education in the clinical control groups) and the forensic context of the clinical samples. Needless to say, the potential impact of demographic variables, such as age, gender, race/ethnicity, and education on SDI scores requires further examination.

The accuracy of the SDI in differentiating factitious from malingered symptom presentations differs noticeably between studies 1 and 2 (AUCs study 1 up to .88; AUCs study 2 up to .75; see also Table 7). A conceivable factor in causing this divergence is the similarity of the situational background that was sketched in the factitious and malingering conditions of study 2: All participants were prompted to imagine having been involved in a quarrel at a college party and an ensuing court case. The malingering condition asked participants to act as if they were still engaged in the court case, whereas the factitious condition required participants to suppose that the court case had been dismissed and that the psychological evaluation they were about to undergo was unrelated to the case. The experimental materials of the factitious condition of study 1 did not contain any reference to a malingering scenario (court case or otherwise). It could be that some participants in the factitious condition of study 2 did not completely disregard the relevance of the court case and thus simulated less factitious and more malingered symptom presentations as compared with participants in the factitious condition of Study 1.

The novelty of systematic and psychometric approaches to factitious symptom presentations implies that the conclusions derived from the present data are preliminary at best. Nevertheless, we hope they serve to highlight the potential of psychometric assessment of factitious symptom presentations and act as impetus for future research. Particularly encouraging is our finding that the SDI performed on a par with the SIMS in classifying feigned symptomatology, in that this suggests that items gauging internal incentives do not diminish the efficacy of regular SVT items. Other key findings of the present studies pertain to potential detection strategies for internal incentives associated with factitious disorder. In comparison to participants in the malingering conditions, as per the instrument results, participants in the factitious conditions expressed (a) greater willingness to discuss symptomatology outside professional examinations or treatment; (b) higher motivation to undergo disagreeable or distressful treatment or diagnostic procedures; (c) stronger feelings of resentment or dissatisfaction with previous proceedings concerning their alleged symptoms; (d) greater sensitivity and susceptibility to mild somatic symptomatology; and (e) higher levels of distress and anxiety over potential illnesses and health risks. In conclusion, the current studies demonstrate that the assessment of factitious disorder may benefit from psychometric investigations of symptom validity and that psychometric investigations of symptom validity may benefit from detection strategies for internal incentives associated with factitious disorder.
